# A Phase Ib/II, open-label, multicenter study of INC280 (capmatinib) alone and in combination with buparlisib (BKM120) in adult patients with recurrent glioblastoma

**DOI:** 10.1007/s11060-019-03337-2

**Published:** 2019-11-27

**Authors:** Martin van den Bent, Analia Azaro, Filip De Vos, Juan Sepulveda, W. K. Alfred Yung, Patrick Y. Wen, Andrew B. Lassman, Markus Joerger, Ghazaleh Tabatabai, Jordi Rodon, Ralph Tiedt, Sylvia Zhao, Tiina Kirsilae, Yi Cheng, Sergio Vicente, O. Alejandro Balbin, Hefei Zhang, Wolfgang Wick

**Affiliations:** 1 Erasmus University Medical Center (MC) Cancer Institute, Rotterdam, The Netherlands; 2 Molecular Therapeutics Research Unit (UITM), Department of Medical Oncology, Vall d’Hebron University Hospital, Barcelona, Spain; 3 University Medical Center Utrecht, Utrecht, The Netherlands; 4 Hospital Universitario, 12 de Octubre, Madrid, Spain; 5 MD Anderson Cancer Center, Houston, TX USA; 6 Center for Neuro-Oncology, Dana-Farber Cancer Institute and Harvard Medical School, Boston, MA USA; 7 Department of Neurology and Herbert Irving Comprehensive Cancer Center, Columbia University Irving Medical Center, New York, NY USA; 8 Cantonal Hospital, St. Gallen, Switzerland; 9 Interdisciplinary Division of Neuro-Oncology, Center for CNS Tumors, Comprehensive Cancer Center, University Hospital Tübingen, Hertie Institute for Clinical Brain Research & Eberhard Karls University Tübingen, German Cancer Consortium (DKTK), DKFZ Partner Site Tübingen, Tübingen, Germany; 10 Novartis Pharma AG, Basel, Switzerland; 11 Novartis Institutes for Biomedical Research (China), Shanghai, China; 12 Novartis Institutes for Biomedical Research (United States), Boston, MA USA; 13 Clinical Cooperation Unit Neurooncology, German Cancer Consortium (DKTK), German Cancer Research Center (DKFZ), and Neurology Clinic and National Center for Tumor Diseases, University Hospital Heidelberg, Heidelberg, Germany

**Keywords:** Glioblastoma, INC280, Capmatinib, Buparlisib, c-Met, PTEN

## Abstract

**Purpose:**

To estimate the maximum tolerated dose (MTD) and/or identify the recommended Phase II dose (RP2D) for combined INC280 and buparlisib in patients with recurrent glioblastoma with homozygous phosphatase and tensin homolog (PTEN) deletion, mutation or protein loss.

**Methods:**

This multicenter, open-label, Phase Ib/II study included adult patients with glioblastoma with mesenchymal-epithelial transcription factor (c-Met) amplification. In Phase Ib, patients received INC280 as capsules or tablets in combination with buparlisib. In Phase II, patients received INC280 only. Response was assessed centrally using Response Assessment in Neuro-Oncology response criteria for high-grade gliomas. All adverse events (AEs) were recorded and graded.

**Results:**

33 patients entered Phase Ib, 32 with altered PTEN. RP2D was not declared due to potential drug–drug interactions, which may have resulted in lack of efficacy; thus, Phase II, including 10 patients, was continued with INC280 monotherapy only. Best response was stable disease in 30% of patients. In the selected patient population, enrollment was halted due to limited activity with INC280 monotherapy. In Phase Ib, the most common treatment-related AEs were fatigue (36.4%), nausea (30.3%) and increased alanine aminotransferase (30.3%). MTD was identified at INC280 Tab 300 mg twice daily + buparlisib 80 mg once daily. In Phase II, the most common AEs were headache (40.0%), constipation (30.0%), fatigue (30.0%) and increased lipase (30.0%).

**Conclusion:**

The combination of INC280/buparlisib resulted in no clear activity in patients with recurrent PTEN-deficient glioblastoma. More stringent molecular selection strategies might produce better outcomes.

**Trial registration**: NCT01870726.

**Electronic supplementary material:**

The online version of this article (10.1007/s11060-019-03337-2) contains supplementary material, which is available to authorized users.

## Introduction

Glioblastomas are the most common type of brain tumor and generally have a limited response to available therapies [1]. Even when optimally managed with combined chemo-irradiation, patients with glioblastomas have poor outcomes [2] with a median survival of 14–16 months in study cohorts. Available options for recurrent or progressive tumors are limited and novel therapeutic options are urgently needed. Glioblastoma growth is driven by aberrant activity of one or more signaling pathways. Dysregulation of the proto-oncogene MET (c-Met), and the phosphatidylinositol 3-kinase (PI3K) signaling pathways are frequent in glioblastoma [3, 4]. Preclinical and translational studies have indicated that activation of MET and PI3K signaling are important in tumor initiation and maintenance [5]. Inhibition of MET can have potent anti-tumor effects, including regression of human glioblastoma tumor xenografts [6, 7]. Loss of phosphatase and tensin homolog (PTEN), a negative regulator of PI3K, by mutation or gene deletion is the most common form of PI3K pathway dysregulation, occurring in around 25–44% of all glioblastomas [3, 8]. With complex genetic alterations in glioblastomas, blocking only one pathway may be insufficient to fully impede cancer cell growth, thus, combining therapies that strategically target multiple pathways may improve clinical outcomes in patients who fail first- or second-line treatment for recurrent glioblastoma.

In preclinical models, buparlisib (BKM120), a PI3K inhibitor, has been combined with INC280 (capmatinib), a MET inhibitor, and synergy between the two agents has been observed in PTEN-null glioblastoma cell lines that express hepatocyte growth factor (HGF; data not shown). In addition, in an in vivo model of a human glioblastoma xenograft with a PTEN mutation and HGF expression (presumably leading to autocrine MET activation), the combination of these two agents was significantly more efficacious than either agent alone (Supplemental Fig. 1). INC280 has also demonstrated preclinical and clinical activity in tumors with MET dysregulation [9–12]. Buparlisib has demonstrated activity in tumors with PI3K activation [13–15].

**Fig. 1:**
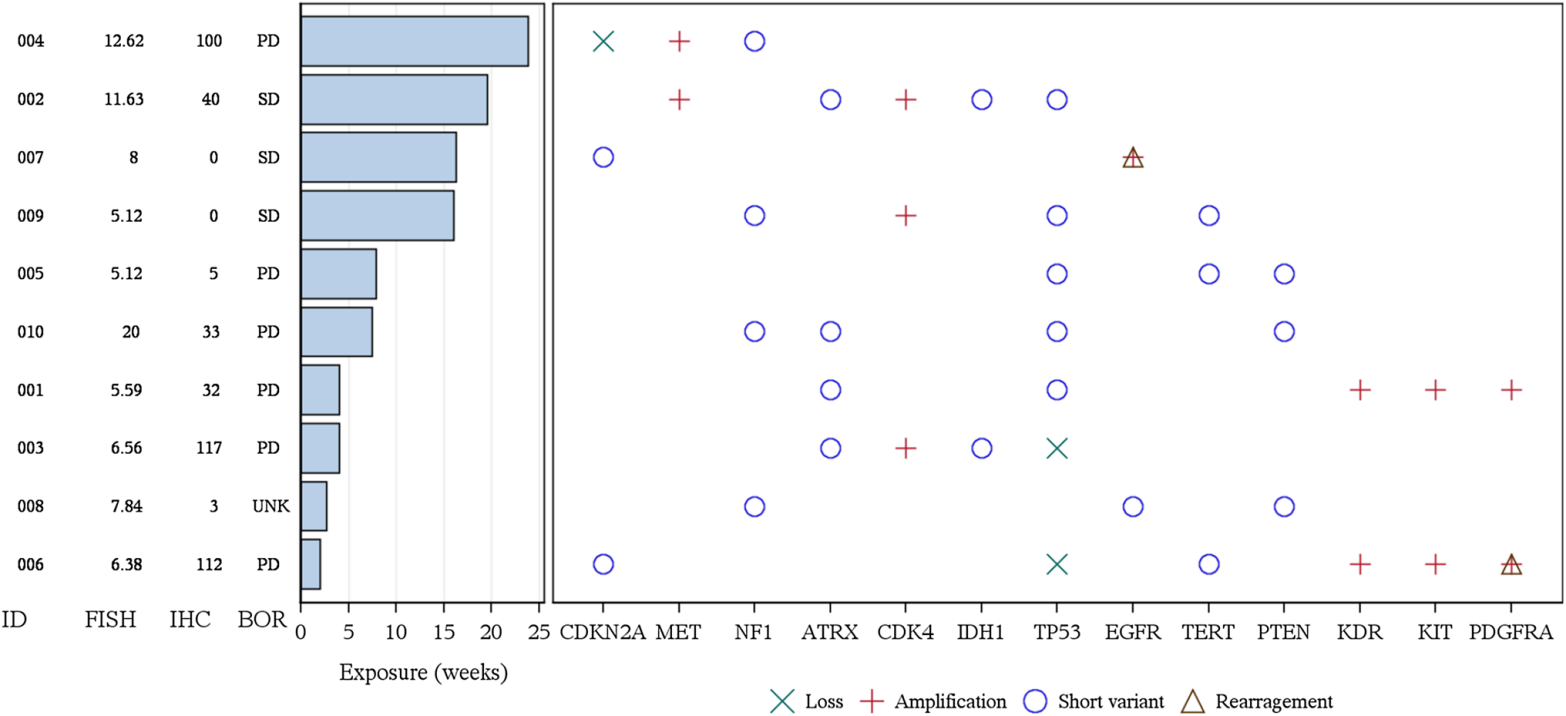
Most frequent somatic genetic alterations observed in tumor samples with known/likely functional significance using Next Generation Sequencing analysis and duration of exposure. Phase II subjects only; two (or more) alterations were observed with known/likely functional significance; *ATRX*, ATP-dependent helicase ATRX, *BOR* best overall response, *CDK4* cyclin dependent kinase 4, *CDKN2A* cyclin-dependent kinase inhibitor 2A, *EGFR* epidermal growth factor receptor, *FISH* fluorescent in situ hybridization (for MET gene copy number in the nuclei), *ID* patient identification number, *IDH1* isocitrate dehydrogenase 1, *IHC* immunohistochemical staining score (of MET protein expression at the plasma membrane or in the cytoplasm), *KDR* kinase insert domain receptor, *KIT* receptor tyrosine kinase protein KIT, *MET* tyrosine-protein kinase MET, *NF1* neurofibromatosis type 1, *PD* progressive disease, *PDGFRA* platelet-derived growth factor receptor alpha, *PTEN* phosphatase and tensin homolog, *TERT* telomerase reverse transcriptase, *SD* stable disease, *TP53*, tumor protein p53, *UNK* unknown

Here we report results from a multicenter, open-label, Phase Ib/II study. The aim of the Phase Ib part was to estimate the maximum tolerated dose (MTD) and/or to identify the recommended Phase II dose (RP2D) for the combination of INC280 and buparlisib, followed by the Phase II part to assess the clinical efficacy of INC280 as a single agent and in combination with buparlisib, and to further assess the safety.

In addition, a surgical arm (which comprised patients that were candidates for surgical resection) was planned to determine the pharmacokinetic/pharmacodynamic (PK/PD) profile of the study drug combination in patients undergoing tumor resection for recurrent glioblastoma after 7 to 10 days of treatment. Because the RP2D for the combination was not declared, the Phase II was conducted with INC280 monotherapy only.

## Materials and methods

### Study design and conduct

For the Phase Ib part, adults (≥ 18 years) with recurrent glioblastoma and documented homozygous PTEN deletion, PTEN mutation or protein loss assessed with immunohistochemistry (IHC) for PTEN (H score < 10) were eligible for enrollment in this study, which was confirmed by local documentation or central assessment. For the Phase II part, patients were pre-screened for MET verified centrally by fluorescence in situ hybridization (FISH) first and, if the gene copy number (GCN) was > 5, were allocated to the INC280 single agent arm. Patients with tumours harbouring fusion transcripts or mutant MET were eligible after documented agreement with the sponsor. Patients with GCN ≤ 5 were pre-screened for PTEN and were planned to be allocated to the combination arm of INC280 with buparlisib (although this arm was never activated; Supplemental Fig. 2).

Phase I single agent trials have determined the MTD and RP2D of buparlisib to be 100 mg/day [16, 17]. Additional key inclusion criteria were Eastern Cooperative Oncology Group performance status (ECOG PS) ≤ 2; histologically confirmed glioblastoma regardless of IDH status, radiologically proven relapse according to the Response Assessment in Neuro-Oncology (RANO) criteria [18], ≤ 2 prior systemic therapies; prior treatment with vascular endothelial growth factor (VEGF) directed therapy was allowed. Key exclusion criteria included pregnancy, prior/current treatment with MET inhibitor or HGF-targeted therapy, prior/current PI3K inhibitors, or mammalian target of rapamycin (mTOR) inhibitors, active cardiac disease or other cardiac abnormalities, gastrointestinal disease or impairment that could significantly alter drug absorption, and history of psychological impairment.

In the Phase Ib part, patients were enrolled into one of six dosing cohorts based on human safety, PK and preclinical PK-efficacy data to receive INC280 as either oral capsules (Cap) or tablets (Tab). The film-coated Tab formulation was developed to improve patient convenience and consequently, compliance. The Tab formulation provides higher exposure than the Cap; the Tab dose was calculated to achieve a comparable exposure rate to the INC280 Cap. Dose escalation of the combination treatment was guided by a Bayesian logistic regression model (BLRM) in order to monitor patient safety. The switch from INC280 Cap to Tab occurred at the start of cohort 5.

The protocol was amended during Phase Ib to allow for a change in local pre-screening to be performed during the dose escalation; a threshold for PTEN negativity of an H score < 10 for PTEN IHC was introduced to align with the threshold currently used by the central laboratory that was based on the medical literature [19].

All patients in Phase II received INC280 monotherapy over a 28-day cycle. Treatment continued until unacceptable toxicity, disease progression or discontinuation at the discretion of the Investigator, or by withdrawal of patient consent. Dose adjustments were permitted to manage treatment-related toxicities.

A protocol amendment was also made during Phase II of the study in which an INC280-monotherapy arm was introduced to investigate single-agent INC280 in glioblastoma patients with altered MET (amplified GCN > 5, fusion or mutant). The inclusion criterion was modified to add ‘MET amplification by FISH (fusion transcripts or mutant MET may be eligible after discussion with the sponsor)’.

### Primary objectives

The primary objective of Phase Ib was to establish the MTD and to identify the RP2D for the combination of INC280 and buparlisib. The primary objective of Phase II was to assess the clinical efficacy and safety of INC280 alone and in combination with buparlisib; and for the surgical arm, the objective was to determine the PK/PD characteristics of the combination of INC280 and buparlisib. This analysis was not performed, as the Phase II part was limited to INC280 monotherapy in MET-amplified glioblastoma based on PK findings of Phase Ib.

### Assessments

Tumor response and progression was assessed using the RANO Working Group response criteria for high-grade gliomas [18]. The radiological evaluation was reviewed centrally. Magnetic resonance imaging and clinical presentation were evaluated at baseline and repeated at 8-week intervals during the study until disease progression, the start of another antineoplastic treatment, or death.

All adverse events (AEs) were recorded and graded according to the Common Terminology Criteria for AEs (CTCAE) version 4.03 at every visit. AE monitoring continued for at least 30 days following the last dose of study treatment. Complete physical examinations and assessment of vital signs were performed on scheduled days. When dose-limiting toxicity (DLT) occurred, study treatment was interrupted and the toxicity was managed according to pre-specified criteria. Blood samples were collected for INC280 and buparlisib PK analysis.

## Results

### Patient characteristics

#### Patient demographics and baseline characteristics

In Phase Ib, patients (n = 33) were primarily male (72.7%), Caucasian (87.9%), with a median age of 59.0 years (Table 1). All except one patient had altered *PTEN* (deletion, mutation or protein loss). One patient had PTEN-positive IHC and no PI3K mutations, but was included based on detection of MET amplification by the investigator’s institution. Patients were entered into one of the following dose cohorts: INC280 Cap 200, 400, or 500 mg twice daily (BID) + 50 mg buparlisib once daily (QD); INC280 Cap 500 mg BID + 80 mg buparlisib QD; or INC280 Tab 300 or 400 mg BID + 80 mg buparlisib QD.

**Table 1:** Patient demographics and baseline characteristics

Characteristic	Phase Ib INC280 + buparlisib (all patients)	Phase II INC280 400 mg BID Tab
N	33	10
Median age, years (range)	59.0 (27–75)	48.0 (32–63)
Sex: male, n (%)	24 (72.7)	3 (30.0)
Race, n (%)		
Black	1 (3.0)	0
Caucasian	29 (87.9)	9 (90.0)
Other	3 (9.1)	0
Unknown	0	1 (10.0)
ECOG PS, n (%)		
0	13 (39.4)	3 (30.0)
1	18 (54.5)	5 (50.0)
2	2 (6.1)	2 (20.0)
Type of last antineoplastic therapy, n (%)		
Medication	27 (81.8)	7 (70.0)
Radiotherapy	1 (3.0)	1 (10.0)
Surgery	5 (15.2)	2 (20.0)

Ten patients entered the INC280 monotherapy arm (Phase II) (median age 48 years; 70% women, 90% Caucasian; Table 1). From 1st June 2015, 148 patients were screened for entry into the Phase II of this study; 10 patients (6.76%) were treated.

#### Biomarkers

In Phase II, patients had a range of MET gene copy number and co-occurring genetic alterations assessed by next generation sequencing (NGS using the Foundation Medicine T7 panel; summarized in Table 2). Further analysis of MET copy number status by NGS in 9 of the 10 Phase II patients revealed that 7/9 showed broad copy number gain of a chromosomal region containing the MET gene, with copy numbers in the range of 4 to 6. Only 2/9 tumors (Patients 002 and 004) showed evidence for focal amplification of the MET gene, with copy number ≥ 9. In line with these observations, the two tumors with focal MET amplification displayed a MET:CEP-7 ratio in the FISH assay of around 5. This ratio was lower (average ~ 1.7) in the 7 tumors with broad copy number gain, with the exception of one tumor (Patient 010) with a marked discrepancy between copy number by FISH and NGS (20 vs. 4 without any evidence of focality in either case). Despite the selection of MET FISH copy number ≥ 5, MET protein expression, as assessed by IHC, was relatively low across tumor samples (Fig. 1). The range of MET gene copy numbers and genetic alterations in the Phase Ib is shown in Table 3.

**Table 2:** NGS data with potential (known or likely) functional significance (Phase II data)

Patient ID	Best overall response	IHC	FISH		FM NGS		Sequencing data with potential (known or likely) functional significance		
			MET copy number	Ratio^b^	MET Copy Number	Ratio^c^	Copy number variant (copy number)	Short variant	rearrangement
001	PD	32	5.59	1.19	5	612.1	KDR(13), KIT(13), PDGFRA(13)	ATRX, EPHA6, H3F3A, HSP90AA1, TP53	N/F
002	SD	40	11.63	4.86	9	1.0	CDK4(95), IGF1R(10), MET(9)	ATRX, IDH1, TP53	N/F
					14^d^	0.4^d^			
003	PD	117	6.56	2.51	5	154.1	CDK4(63),GLI1(22), MYCN(35), TP53(0)	ATRX, IDH1	N/F
004	PD	100	12.62	5.21	16	1.5	CDKN2A(0), CDKN2B(0), MET(18)	AR, NF1, NPM1, PIK3R1, PRDM1	N/F
005	PD	5	5.12	1.26	3	1258.1		PTEN, TERT, TP53	KEAP1
006	PD	112	6.38	1.85	6	63.5	KDR(10), KIT(11), PDGFRA(40), TP53(0)	CDKN2A, FANCL, LZTR1, PIK3CA, TERT	PDGFRA
					6^d^	29.4^d^			
007	SD	0	8	1.41	4	396.3	EGFR(107)	ARAF, BCL2, CDKN2A	EGFR
008	UNK^a^	3	7.84	3.27	N/A	N/A		EGFR, NF1, PTEN	N/F
009	SD	0	5.12	1.08	6	1258.1	CDK4(29)	KMT2C, NF1, TERT, TP53	N/F
010	PD	33	20^e^	2.5	4	1205.6	N/F	APC, ATRX, NF1, PTEN, RB1, TP53	FAT1

*FISH* fluorescent in situ hybridization (for MET gene copy number in the nuclei), *FM* foundation medicine, *ID* patient identification number, *IHC* immunohistochemical staining score, H score (of MET protein expression at the plasma membrane or in the cytoplasm), *N/A* not applicable, *N/F* no findings, *PD* progressive disease, *SD* stable disease, *UNK* unknown

^a^Clinical PD, the lesions were not assessed

^b^Ratio of MET copies to CEP7 copies

^c^Ratio of the size of genomic fragment overlapping with MET relative to the size of the MET gene

^d^Two different segments overlapping the MET gene were called by the analysis pipeline downstream of the hybridization capture and NGS process [31]

^e^Note discrepancy and high copy number by FISH which does not correlate with NGS data and may represent a potential technical issue with FISH

**Table 3:** NGS data with potential (known or likely) functional significance (Phase Ib data)

Patient ID	Best overall response	IHC	Sequencing data with potential (known or likely) functional significance		
			Copy number variant (copy number)	Short variant	Rearrangement
101	PD	0			
102	PD	50	CDK4 (78), GLI1 (18), MDM2 (70), SOX2 (7)	PTEN, TERT	
103	PD		CDK4 (36), MDM2 (65)	PTEN, TERT	
104	PD	80	CDKN2A (0), CDKN2B (0), EGFR (128)	AXL, EGFR, FLT4, KDM5A, TERT	
105	PD	90	EGFR (61), ERRFI1 (0)	EGFR, PTEN, TERT	CDKN2A, EGFR
106	PD	100			
107	PD			ARID1A, FGFR2, PTEN, STAG2	
108	PD		CDKN2A (0), CDKN2B (0), EGFR (110)	EGFR, FAT1, NOTCH1, PTEN, SPTA1, TERT	EGFR
109	PD	101	CDKN2A (0), CDKN2B (0)	FGFR4, NF1, PTEN, RB1, TERT, TP53	NF1
110	PD	100	CDKN2A (0), CDKN2B (0), EGFR (59)	EGFR, PTEN, TERT	
111	PD		CDKN2A (0), CDKN2B (0), EGFR (46), MDM4 (28), PIK3C2B (30)	EGFR, PTEN, TERT	EGFR
112	PD	90	RB1 (0)	NF1, PTEN, TERT	
113	PD	50	CDKN2A (0), CDKN2B (0), MDM4 (53), PIK3C2B (54)	BRCA2, PTEN, STAG2, TERT	EGFR
114	UNK	80	CDKN2A (0), CDKN2B (0), EGFR (40)	PTEN, TERT	
115	PD		CDKN2A (0)	PTEN	
116	PD	100	CDKN2A (0), CDKN2C (0), KDR (6), KIT (6), PDGFRA (6), PTEN (0)	TERT, TP53	
117	PD	110		NF1, PIK3CA, PTEN, RB1, TP53	
118	PD		TP53 (0)	PTEN, TERT	
119	PD	0	CDKN2A (0), CDKN2B (0), EGFR (60)	TERT	
120	PD	0	CCND2 (45), CDK4 (47), EGFR (16), FGF23 (10), FGF6 (10), FRS2 (102), MDM2 (93)	PTEN, TERT	
121	PD	0	CDKN2A (0), CDKN2B (0), EGFR (45)	LRP1B, PTEN, TERT	EGFR
122	PD	55			
123	PD	80	CDKN2A (0), CDKN2B (0), EGFR (92)	EGFR, TERT	EGFR
124	PD	100	CDKN2A (0), CDKN2B (0)	PTEN, STAG2, TERT	
125	PD	30		STK11, TERT	
126	PD				
127	PD	65		PTEN, TERT	
128	SD*	100	CDK4 (61), KIT (6), PDGFRA (6)	TP53	
129	PD	100	CDKN2A (0), CDKN2B (0), EGFR (125)	EGFR	EGFR
130	UNK				
131	PD	90	CDKN2A (0)	BCOR	
132	PD	100	CDKN2A (0), CDKN2B (0), EGFR (72)	EGFR, GLI1, PTEN, TERT	
133	UNK	0	CDKN2A (0), CDKN2B (0), EGFR (42), JUN (9), PTEN (0)	EGFR, TERT	EGFR

*FISH* fluorescent in situ hybridization (for MET gene copy number in the nuclei), *FM* foundation medicine, *ID* patient identification number, *IHC* immunohistochemical staining score, H score (of MET protein expression at the plasma membrane or in the cytoplasm), *PD* progressive disease, *SD* stable disease, *UNK* unknown

^a^Clinical PD, the lesions were not assessed

^b^Ratio of MET copies to CEP7 copies

^c^Ratio of the size of genomic fragment overlapping with MET relative to the size of the MET gene

^d^Note discrepancy and high copy number by FISH which does not correlate with NGS data and may represent a potential technical issue with FISH

^*^Patient achieved stable disease (SD) at Cycle 1, Day 15; by Day 27 of Cycle 1, this patient was assessed to have progressive disease (PD)

### Safety

#### Phase Ib dose escalation

All 33 patients in Phase Ib discontinued study treatment and reported at least one AE. The main reason for study discontinuation was disease progression (n = 29, 87.9%); other reasons were AEs (n = 2) and consent withdrawal/patient decision (n = 2). Treatment-related AEs were reported in 84.8% of the Phase Ib patients. The most commonly reported treatment-related AEs were fatigue (36.4%), nausea (30.3%), alanine aminotransferase increased (30.3%), aspartate aminotransferase increased (24.2%), depression (24.2%) and hyperglycemia (21.2%). Grade ≥ 3 AEs were reported in 24 patients (72.7%). Treatment-related grade ≥ 3 AEs were reported in 12 patients (36.4%). MTD was identified at INC280 Tab 300 mg BID + buparlisib 80 mg QD, a dosage received by 7 patients. DLT was observed in four patients: nausea (INC280 Tab 300 mg BID + buparlisib 80 mg QD; grade 3), personality change (INC280 Cap 400 mg BID + buparlisib 50 mg QD; grade 3), and elevated transaminases in two patients (both INC280 Tab 400 mg BID + buparlisib 80 mg QD; grade 3; Table 4).

**Table 4:** Dose-limiting toxicities by dose

Cohort	Total daily doses INC280 (BID) + buparlisib (QD)	No. of patients treated	No. of patients in the dose-determining set	No. of DLTs in cycle 1
INC280 capsule formulation				
1	200 mg + 50 mg	5	4	0
2	400 mg + 50 mg	6	5	1
3	500 mg + 50 mg	4	3	0
4	500 mg + 80 mg	6	4	0
INC280 tablet formulation				
5	300 mg + 80 mg	7	7	1
6	400 mg + 80 mg	5	4	2

*BID* twice daily, *DLT* dose limiting toxicity, *QD* once a day

#### Phase II

As in Phase Ib, all patients in Phase II reported at least one AE. Treatment-related AEs were reported in 60.0% of the Phase II patients. The most commonly reported AEs by preferred term were headache (40.0%), constipation (30.0%), fatigue (30.0%) and increased lipase (30.0%). Grade ≥ 3 AEs were reported in nine patients (90.0%). Treatment-related grade ≥ 3 AEs were reported two patients (20.0%).

In terms of exposure to INC280, the average mean daily dose (± standard deviation, SD) for all patients in the Phase II part of this study was 754.1 mg (± 125.21), with a cumulative dose of 54,220.0 mg (± 43,045.24).

### Pharmacokinetics

During Phase Ib, the target exposures for both drugs in the combination therapy were not met in the combination treatment arm. Compared with data from single-agent treatment studies, the exposures of INC280 and buparlisib were significantly lower when dosed in combination (Table 5). Compared with single-agent INC280 (CINC280A2201, data on file), the area under the curve (AUC) of INC280 400 mg BID in combination with buparlisib 80 mg QID was 0.73-fold. Compared with single-agent buparlisib [17], the AUC of buparlisib 80 mg QD in combination with INC280 400 mg BID was 0.38-fold. The mechanism for this reduced exposure is not known at present but the possibility of drug–drug interaction cannot be ignored. AUCs and other pharmacokinetic parameters are presented in Table 5.

**Table 5:** Primary pharmacokinetic parameters for INC280 and for buparlisib (Phase 1b data)

Cycle 1, Day 15	INC 200 mg Cap BID + bup 50 mg QD	INC 400 mg Cap BID + bup 50 mg QD	INC 500 mg Cap BID + bup 50 mg QD	INC 500 mg Cap BID + bup 80 mg QD	INC 300 mg Tab BID + bup 80 mg QD	INC 400 mg Tab BID + bup 80 mg QD
INC280						
N	5	4	3	2	3	4
AUC_tau_ (h*ng/mL)	6260	8580	12,800	2650	12,200	15,300
Geo-mean (Geo-CV%)	(45)	(79)	(99)	(46)	(33)	(19)
C_max_ (ng/mL)	1350	1850	3400	494	3460	3870
Geo-mean (Geo-CV%)	(59)	(78)	(114)	(71)	(39)	(55)
Buparlisib						
N	5	5	3	2	4	4
AUC_tau_ (h*ng/mL)	8210	5190	6270	10,047	9950	7180
Geo-mean (Geo-CV%)	(33)	(50)	(18)	(61)	(13)	(39)
N	5	5	3	2	5	4
C_max_ (ng/mL)	680	429	580	779	853	799
Geo-mean (Geo-CV%)	(13)	(38)	(29)	(19)	(28)	(67)

Geometric mean AUCtau, ss of INC280 tablet 400 mg bid is 21,000 ng*hr/mL in monotherapy (INC280 IB v6);

Geometric mean AUCtau, ss of buparlisib 80 mg qd is 19,100 ng*hr/mL in monotherapy (BKM120 IB v10)

Vertical, heavy line indicates the INC280 Cap vs Tab treatments

*AUC* area under the curve, *BID* twice daily, *bup* buparlisib, *cap* capsule, *Cmax* maximum (peak) observed drug concentration, *INC* INC280, *QD* once daily, *tab* tablet

### Efficacy

#### Overall efficacy

The combination of INC280 + buparlisib demonstrated very limited activity in these 33 patients with PTEN-altered glioblastoma. RP2D was not declared due to potential drug–drug interactions and hence a low drug exposure, which may have resulted in lack of observed efficacy with the INC280 and buparlisib drug combination in Phase Ib. Consequently, the combination arms planned for Phase II were not initiated.

In the Phase II INC280 monotherapy arm, 10 patients were enrolled. No patient achieved partial (PR) or complete response (CR). Best response of stable disease (SD) was observed in 3 of 10 patients (30.0%) in Phase II, and lasted between 16–20 weeks from the start of treatment until disease progression, similar to the exposure time (Fig. 1). Due to the limited activity observed with INC280 monotherapy (400 mg BID Tab) in this population of patients with recurrent glioblastoma, the enrollment of patients was halted early after pre-planned futility analysis and the primary endpoint, progression-free survival rate at 6 months, was not assessed due to insufficient sample size.

#### Efficacy according to biomarkers

All alterations identified and key co-occurring genetic alterations as identified by NGS are shown in Table 2. Alterations in several genes previously linked to glioblastoma (e.g. PTEN, TP53, EGFR) [3] were detected, along with other mutations of unknown significance.

## Discussion

This study was initially based on the hypothesis that INC280 and buparlisib would have a synergistic anti-tumor activity in recurrent glioblastoma with concomitant MET and PI3K activation. The safety profile of the combination of INC280 and buparlisib was consistent with the known safety profile of these agents as monotherapies in the oncology setting [10, 11, 20–22] No new safety signals were identified. One patient experienced a personality change, which is consistent with the know safety profile of buparlisib [20]. RP2D was not declared due to a lack of efficacy in the drug combination, low drug exposure and potential drug–drug interactions in the Phase Ib stage of this trial.

During the conduct of this trial, INC280 film-coated Tabs were introduced into the study to improve patient convenience, based on a relative bioavailability study (data on file) in which INC280 Tabs were shown to provide higher drug exposure than Caps.

Originally, it was not thought that INC280 or buparlisib would have sufficient single-agent activity to block cancer cell growth due to the complex genetic alterations in glioblastoma. However, while Phase Ib of this trial was in progress, INC280 showed preliminary efficacy signals in two patients with MET amplified recurrent glioblastoma in other trials (unpublished data on file and a patient receiving compassionate use of INC280 + inhibitor LDE225). Additionally, INC280 has shown promising clinical efficacy in non-small-cell lung carcinoma with MET amplification [10]. Based on this emerging clinical evidence, the decision was made to continue with a monotherapy arm only in Phase II to investigate single-agent INC280 in MET amplified glioblastoma patients. For this part of the study, patients were enrolled if their tumors showed a relative MET copy number of ≥ 5, as measured using a FISH assay.

No evidence of activity was observed with INC280 monotherapy in Phase II. However, the majority of Phase II patients had tumors with elevated MET copy number in the context of broad gain of chromosome 7. In addition, MET protein expression in those tumor samples, as measured by IHC, was relatively low despite increased MET gene copy numbers (Table 2).

The discrepancy between MET copy number (FISH) and protein expression (IHC) is one that requires careful consideration and highlights the challenges of defining molecular inclusion criteria for clinical trials. Given the small sample size it is difficult to determine the cause of the apparent discrepancy between gene copy number and protein expression. Several possible explanations exist. Sample age may have played a role in the low IHC results as all samples were from archival material (mean [SD] sample age for Phase II of 514 [± 359] days). Discordance between FISH and IHC has been described before for other cancers [23–26]. Moreover, simple chromosome polysomy does not necessarily lead to increased transcription.

Significant heterogeneity regarding co-occurring genetic alterations was observed across the 10 patients with presumed MET amplification (Table 2). The detected alterations are consistent with the previously described glioblastoma landscape [3].

Recent and ongoing trials of INC280 in lung cancer and hepatocellular carcinoma are exploring the predictive markers that are suggested by preclinical data. So far, MET exon 14 skipping mutations in lung cancer are emerging as the most robust predictive marker, and the clinical data suggest that both MET copy number and protein overexpression may have predictive value as well, but appropriate cut-offs still need to be established [10, 27, 28] MET exon 14 skipping mutations have also recently been reported in secondary glioblastoma with a frequency of ~ 14%, and at lower frequencies in primary glioblastoma and low-grade glioma [29]. In addition, PTPRZ1-MET fusions were found in secondary glioblastoma, where they can co-occur with MET exon 14 skipping mutations [7, 29]. PTPRZ1-MET and other MET fusions were also reported in pediatric glioblastoma [30]. Preclinical as well as emerging clinical data suggest that brain malignancies with MET mutations and/or fusions are responsive to MET inhibitors [29, 30]. Therefore, optimizing patient selection for investigation of INC280 in glioblastoma may require a more comprehensive characterization of MET molecular abnormalities beyond copy number. Another potential predictive biomarker that should be considered in future trials of MET inhibitors in glioblastoma is HGF expression by the tumor, based on preclinical data [12]. While there is good rationale for targeting MET in glioblastoma, our study illustrates the need for further molecular profiling to identify the subset of patients who may benefit.

INC280 has shown some degree of brain penetration in preclinical species (our unpublished observation), but the extent of brain exposure and MET inhibition in patients with glioblastomas are unknown and may also have affected outcome. Future trials on novel agents should study this systematically early on in the clinical trial program to ensure the target is reached.

To conclude, the combination INC280/buparlisib resulted in reduced exposure of both drugs and no clear signal of activity in recurrent PTEN-deficient glioblastoma. With the assay and cut-off for MET amplification used, no clear activity signal was seen with INC280 single-agent treatment. However, consideration of confounding factors and a more stringent molecular selection strategy could be used to further explore the role of MET inhibitors for the treatment of recurrent glioblastoma.

## Study limitations

This study is limited by the lack of data available on the MET GCN cut-off number for molecular selection. We used ≤ 5 as a cut-off based on limited emerging data from other capmatinib trials, and, due to the relatively small and potentially molecularly diverse patient population, we were unable to refine this copy number in the current study. This molecular-based therapy uses ‘historical information’ because all biopsies to determine MET status were archival, without accounting for the effects of intervening therapy or molecular drift. Thus, it is possible the molecular profile at study entry differed from that extrapolated from the analysis of archival tissue.

## Electronic supplementary material

Below is the link to the electronic supplementary material.

Supplementary file1 (DOCX 161 kb)
