# Supplementary material for: A Phase Ib/II, open-label, multicenter study of INC280 (capmatinib) alone and in combination with buparlisib (BKM120) in adult patients with recurrent glioblastoma
Source: J Neurooncol. 2019 Nov 27;146(1):79–89. doi: 10.1007/s11060-019-03337-2 (PMC6938467; doi:10.1007/s11060-019-03337-2)
Supplement: Supplementary file 1 — Supplementary file1 (DOCX 161 kb) [file 11060_2019_3337_MOESM1_ESM.docx]

**Supplemental Figure 1**. U-87 MG xenograft study


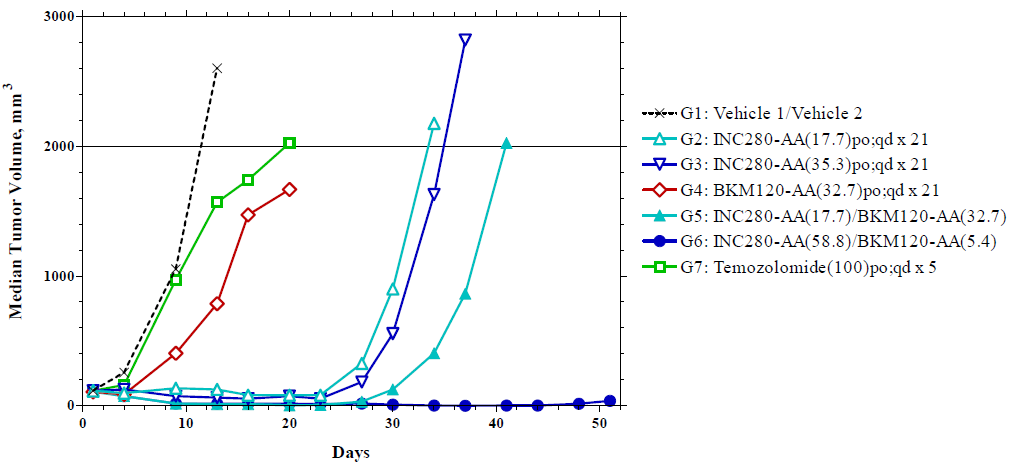


Median tumor volumes of U-87 MG xenografts in the indicated treatment groups (G1 to G7). AA signifies the hydrochloride salt of the respective compound. Numbers in parentheses indicate the dose in mg/kg of the respective compound salt; po, administration by oral gavage; qd, once daily; x 21 or x 5 indicate that treatments lasted for 21 or 5 days, respectively; combination treatments lasted for 21 days. Vehicle 1 = 0.25% methylcellulose and 0.05% Tween® 80 in deionized water, Vehicle 2 = 0.5% methylcellulose and 0.5% Tween® 80 in deionized water. Animals were euthanized when their xenograft reached the endpoint volume (2000 mm^3^), or on the last day of the study (D51).

Because of rapid tumor progression in some of the treatment groups, short-term activity was analyzed on D13. Efficacy was determined as T/C (%), the percent mean tumor volume change from D1 to D13 in drug-treated (ΔT) versus vehicle-treated (ΔC, 2512 mm^3^) mice, or as T/T0, the group mean reduction in tumor volume from D1. A T/C ≤ 40% generally indicates potential therapeutic activity. All drug-treated groups were compared to the control group with Kruskal-Wallis analysis and Dunn’s post hoc multiple comparison test; these tests were repeated to compare the low-dose INC280 combination to its corresponding monotherapies. Temozolomide at 100 mg/kg produced 48% T/C, and did not achieve significant inhibition in this analysis. INC280 monotherapies at 17.7 and 35.3 mg/kg resulted in weakly dose-dependent 5% and 1% T/C, and significant median growth inhibition (P < 0.01 and P < 0.001), respectively. BKM120 monotherapy at 32.7 mg/kg resulted in 31% T/C, and non-significant inhibition. Both combinations yielded significant median activity (P < 0.001). INC280/BKM120 at the 17.7:32.7 mg/kg dose ratio produced –83% T/T0, and significantly outperformed the corresponding INC280 (P < 0.05) and BKM120 (P < 0.001) monotherapies. INC280/BKM120 at the 58.8:5.4 mg/kg ratio produced –75% T/T0; no monotherapies corresponded to this combination.

The study was conducted in compliance with the recommendations of the Guide for Care and Use of Laboratory Animals with respect to restraint, husbandry, surgical procedures, feed and fluid regulation, and veterinary care. The animal program at the CRO that conducted the experiment (Piedmont Research Center) is accredited by the Association for Assessment and Accreditation of Laboratory Animal Care (AAALAC) International, which assures compliance with accepted standards for the care and use of laboratory animals. More details on the study are on file and available upon request.

**Supplemental Figure 2**. Study design


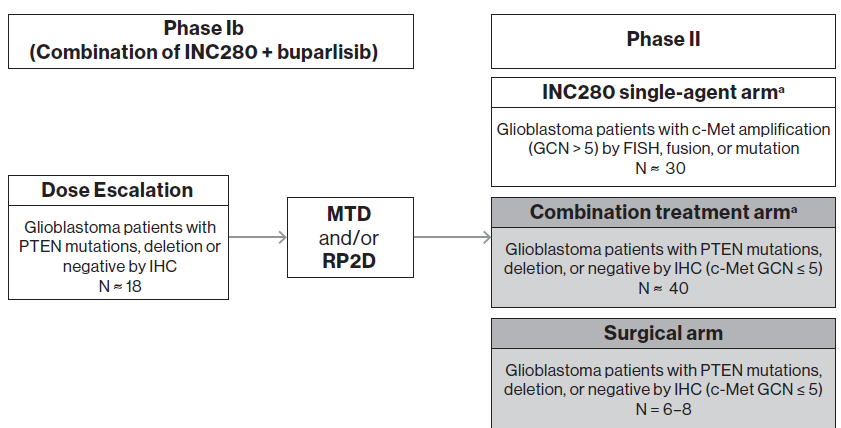


GCN, gene copy number; FISH, fluorescent *in situ* hybridization; IHC, immunochemistry; MTD, maximum tolerated dose; RP2D, recommended Phase II dose

^a^The single-agent arm was added in an amendment to this study

*Phase II arms shown in grey were not enrolled
